# Comparing bee species responses to chemical mixtures: Common response patterns?

**DOI:** 10.1371/journal.pone.0176289

**Published:** 2017-06-22

**Authors:** Alex Robinson, Helen Hesketh, Elma Lahive, Alice A. Horton, Claus Svendsen, Agnes Rortais, Jean Lou Dorne, Jan Baas, Matthew S. Heard, David J. Spurgeon

**Affiliations:** 1 Centre for Ecology and Hydrology, Wallingford, Oxon, United Kingdom; 2 European Food Safety Authority, Parma, Italy; Institut Sophia Agrobiotech, FRANCE

## Abstract

Pollinators in agricultural landscapes can be exposed to mixtures of pesticides and environmental pollutants. Existing mixture toxicity modelling approaches, such as the models of concentration addition and independent action and the mechanistic DEBtox framework have been previously shown as valuable tools for understanding and ultimately predicting joint toxicity. Here we apply these mixture models to investigate the potential to interpret the effects of semi-chronic binary mixture exposure for three bee species: *Apis mellifera*, *Bombus terrestris* and *Osmia bicornis* within potentiation and mixture toxicity experiments. In the potentiation studies, the effect of the insecticide dimethoate with added propiconazole fungicide and neonicotinoid insecticide clothianidin with added tau-fluvalinate pyrethroid acaricide showed no difference in toxicity compared to the single chemical alone. Clothianidin toxicity showed a small scale, but temporally conserved increase in exposure conducted in the presence of propiconazole, particularly for *B*. *terrestris* and *O*. *bicornis*, the latter showing a near three-fold increase in clothianidin toxicity in the presence of propiconazole. In the mixture toxicity studies, the dominant response patterns were of additivity, however, binary mixtures of clothianidin and dimethoate in *A*. *mellifera*, *B*. *terrestris* and male *O*. *bicornis* there was evidence of a predominant antagonistic interaction. Given the ubiquitous nature of exposures to multiple chemicals, there is an urgent need to consider mixture effects in pollinator risk assessments. Our analyses suggest that current models, particularly those that utilise time-series data, such as DEBtox, can be used to identify additivity as the dominant response pattern and also those examples of interactions, even when small-scale, that may need to be taken into account during risk assessment.

## Introduction

Widespread agrochemical use and pollution means that foraging bees can be exposed to contaminants singly, sequentially or in a range of combinations [1–3]. Major reviews of the effects of mixtures across a range of species and chemical combinations have suggested that the dominant response pattern in mixture exposures is one of joint effects within a factor of two fold of additive predictions for the majority (approximately 80%) of cases [4, 5]. Even though additive is by far the most commonly reported pattern of mixture response, there are nonetheless existing reports of synergism (i.e. joint toxicity higher than expected based on the default assumption of additivity) and antagonism (joint toxicity lower than expected in relation to assumptions of additivity) chemical mixtures. Among bee species, examples of interactive effects seen include large-magnitude synergisms between tau-fluvalinate (a pyrethroid used for *Varroa destructor* mite control) and different sterol biosynthesis inhibiting fungicides [6]; synergisms between tau-fluvalinate and the organophosphate coumaphos, both used for in hive *V*. *destructor* control [7] and synergisms between neonicotinoids and ergosterol biosynthesis inhibiting fungicides, particularly for cyano-substituted compounds such as thiacloprid and acetamiprid [8]. In all cases, the underlying mechanism of the interaction was associated with inhibition by one compound of the active sites of detoxifying cytochrome P450 enzymes that thereby inhibited the metabolism of the second compound. Further smaller scale synergism (<3 fold maximum magnitude) have also been shown for combinations of neonicotinoids and sterol biosynthesis inhibiting fungicides [9], and for a range of pesticides used in orchards again with sterol biosynthesis inhibiting fungicides [10].

With mixture exposures so ubiquitous in nature, and interactive effects between chemicals previously observed, a number of approaches will be needed to support mixture hazard and risk assessment in bees [11]. The two most established “reference” models for mixture effects are concentration addition (CA) for similarly acting chemicals and independent action (IA) for dissimilarly acting chemicals [12, 13]. These two mathematical concepts can be used within the context of conventional concentration-response analysis. While applicable for many mixtures, there are chemical combinations for which these two reference models may fail to fully describe joint effects due to non-additive interactions [4, 14]. The “MIXTOX” approach of Jonker et al. [15] has been widely used to identify such synergistic, antagonistic, dose ratio and dose level interactions that lead to deviations of effect from CA or IA predictions for single time-points [16]. The Dynamic Energy Budget (DEB) theory approach uses a mechanistic based model for mixture toxicity assessment. DEBtox models integrate the time course of effect data within one consistent framework. This allows joint effects to be interpreted in a toxicokinetic and toxicodynamic framework for each single chemical independently. Inclusion of an interaction parameter can, further, allow for the detection of consistent interactions in mixture exposures [17–19].

As there is a clear need for mixture assessment for pollinators, we here examine the joint effects of binary mixtures of pesticides, and among pesticides and environmental contaminants using MIXTOX and DEBtox approaches for data interpretation. The aim of the work was to investigate a series of binary mixtures to identify examples of additive and interactive joint effects within two different data analysis frameworks across both bee species and exposure times. Bioassays were conducted with combinations of insecticides from different classes, fungicides and also environmental contaminants initially in the European honeybee *Apis mellifera*. The patterns of joint effect observed in this species were then compared with those for the bumblebee *Bombus terrestris* and solitary bee *Osmia bicornis* to assess if the patterns of joint effect seen in *A*. *mellifera* were repeated in other bee species This analysis identified the patterns of joint effects across a series of relevant mixtures and species as an indication of the value and uncertainty associated with the application of available mixture tools for assessing risks to bees.

## Materials and methods

### Chemical selection

Six binary mixture combinations were tested. Each represented a chemical pair to which bees could plausibly be jointly exposed, either via direct contact, oral consumption of contaminated resources (nectar, pollen, guttation water) or through indirect contact with contaminated nest mates, comb or food stores. Different mechanistic categories including similar and dissimilar combinations were also tested. All six mixtures were tested for *Apis mellifera*, with three combinations also tested in *Bombus terrestris* and *Osmia bicornis* (see summary Table 1) for which clear concentration response curves were already available [20, 21]. Mixtures tested included both cases where the two chemicals had an effect on the same physiological effect (e.g. on nerve function for the insecticides), even if action was not mediated by the same molecular initiating event (e.g. acetylcholinesterase binding for dimethoate, nicotinic receptor binding for clothianidin, sodium channel binding for tau-fluvalinate) as well as combinations with different modes of action. Organic chemicals are known to be metabolised in bees by the cytochrome P450 system [7, 22, 23]. Since previous studies have identified that sterol inhibiting fungicides can inhibit such metabolism leading to interactive toxicity [6, 8, 24], a sterol biosynthesis inhibiting fungicide (propiconazole) was included in some mixtures with insecticides. Environmental contaminants are known to affect epigenetic regulation (arsenic) and to suppress metabolic rate (cadmium). The modes of action of these two trace elements represent commonly observed effects for other metals and even organic contaminants. Hence their inclusion extend the range of combinations assessed to include mixtures with clearly differing mechanisms. All chemicals were purchased as high grade technical reagents. Stock solutions for dosing to the sucrose solution food source were prepared by dissolving chemicals in either MilliQ water (clothianidin, dimethoate, Cd, As) or acetone (tau-fluvalinate, propiconazole).

**Table 1:** Summary of the Potentiation experiments that involved tests of the concentration response of the first listed chemicals in the presence and absence (+/-) of the second chemical and mixture toxicity conducted with different exposure levels and ratio of the two chemicals undertaken for *Apis mellifera*, *Bombus terrestris* and *Osmia bicornis*.

*Treatments and mixtures tested*	*Apis mellifera*	*Bombus terrestris*	*Osmia bicornis*
*Potentiation experiments*			
*Dimethoate ± Propiconazole*	X		X
*Clothianidin ± Propiconazole*	X	X	X
*Clothianidin ± Tau-fluvalinate*	X	X	
*Mixture toxicity experiments*			
*Clothianidin & Dimethoate*	X	X	X
*Clothianidin & Cadmium*	X		
*Cadmium & Arsenic*	X		

### Overall experimental designs

The experimental designs used were consistent among species, with the overall choice of design based on whether previous studies (e.g. [20, 21]) showed one or both chemicals to have an effect on survival at the tested concentrations. Controls containing only 50% w/v sucrose were included in all experiments. Further, for some chemicals (tau-fluvalinate, propiconazole) spiking of the sucrose solution with the required chemical concentration had to be conducted using acetone as a solvent carrier due to their low water solubility. When this was the case acetone concentrations were kept to a minimum (<1% acetone in the sucrose solution) and additional acetone controls were included in the overall experiment. All treatments used in the experiments were spiked such that each contained the same amount of acetone as used to spike the highest tested concentration. A treatment containing an estimated 96 h LC_50_ of dimethoate for *A*. *mellifera* and *B*. *terrestris* and a 48 h LC_50_ for *O*. *bicornis* were also included as a positive control in each experiment. Dimethoate concentrations used for these positive controls were 1.17 mg/L for *A*. *mellifera*, 1.3 mg/L for *B*. *terrestris* and 2.41 mg/L for *O*. *bicornis*. Survival in these positive controls across all experimental positive control treatments were 66% for *A*. *mellifera* and 61% for *B*. *terrestris* at 96 h, and 53% for *O*. *bicornis* at 48 h. These survival rates are each broadly consistent with expected sensitivity across all experiments.

**Fig 1:**
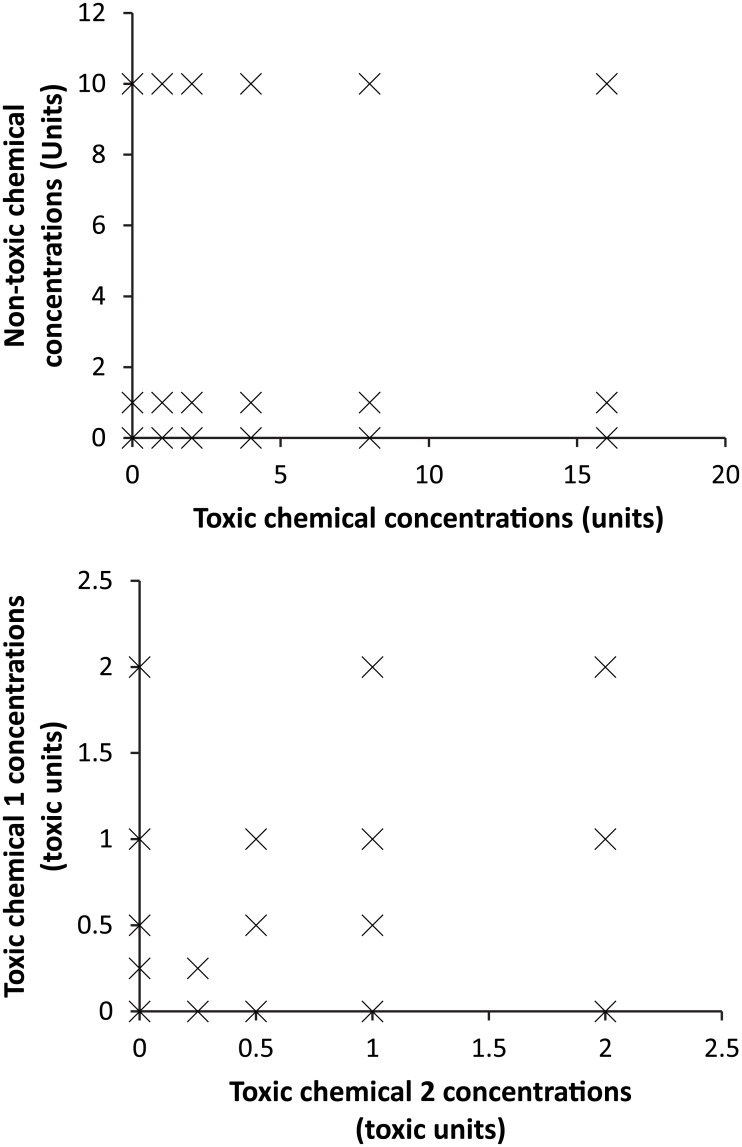
Designs for mixture experiments for cases where only one tested chemical shows a concentration response (A. Potentiation design) and where both chemicals show a concentration response (B. Mixture toxicity design).

The two main types of test designs used were: “Potentiation” experiments and “Mixture Toxicity” experiments (see Table 1). Potentiation experiments were conducted for cases where only one of the chemicals (dimethoate or clothianidin) in the binary mixture was expected to cause adverse effects on survival across tested concentrations. Bees were exposed to a range of concentrations of this toxic chemical in the presence or absence of the second chemical, which was not expected to affect survival at the tested concentrations (design shown in Fig 1a, exact tested concentration for all experiments detailed within the raw data file associated with this work available through Dryad under doi:10.5061/dryad.676ng). From this design, the mixture effect could be assessed as the potentiation (i.e. increase) or alleviation (i.e. decrease) in the effects of the overtly toxic chemical as a result of the presence of the second chemical. The default expectation based on an assumption of no interaction was that the concentration effect responses for the toxic chemical would be similar in each separate series, irrespective of the presence, or not, of the second substance. The concentrations of the potentiating chemicals (tau-fluvalinate or propiconazole) were set at 10x reported environmental concentrations to represent a plausible environmental worst case exposure, given that there was only an extremely small amount of environmental measurement data for each compound available. Reported environmental concentrations are 0.042 mg/L for propiconazole as measured in honey and 0.221 mg/L for tau-fluvalinate in bee bread [25–27]. In some experiments, a further treatment of 100x the environmental concentrations of the potentiating chemical was also included as a toxicological case study. Exposure concentrations of the potentiating chemicals were always tested separately to confirm they had no direct effect on survival.

Mixture Toxicity experiments (see Table 1) included two chemicals each shown to cause overt effects on survival at the tested concentrations [20]. Three mixture toxicity experiments were conducted using a concentration addition (CA) design that would also allow analysis for independent action. Treatments included different effect levels (e.g. 0, 0.25, 0.5, 1 and 2 toxic unit (TU) treatments) and also different mixture ratios (e.g. equitoxic, dominated by compound 1, dominated by compound 2) to assess how effects on survival were affected across the concentration range for different mixtures (design shown in see Fig 1b). Inclusion of single chemical treatments at the same levels as used in the mixture treatments was key to allowing analysis against independent action (IA) model predictions. Thus, the design used allows a robust assessment of the extent to which observed effects relate to predictions based on single chemical effects assuming either similar or dissimilar modes of action. The default expectation based on an assumption of no interaction was of additivity according to prediction of one or more of the CA or IA models.

### Toxicity test protocols

Detailed protocols for testing in each species are provided in Heard et al. [20] and are summarised here. For *A*. *mellifera*, eight hives were established and managed according to standard local bee keeping practices including minimal parasite control and additional feeding to ensure colonies were not stressed by resource limitations early in the season. All bees used were taken from these managed hives. Each test comprised a series of single chemical or mixture treatments, with three hive replicates used for each treatment. Replicates comprised groups of 10 young worker bees harvested from 1–2 brood frames from each hive. Bees were chilled at -20°C for no longer than 45s to allow loading into 0.9 L cages (dimensions height 14.3 cm; width (rim, base) 9.4 cm, 6.7 cm). Individual 50 ml syringe feeders containing 10 ml of appropriately dosed 50% w/v sucrose solution were inserted through a hole in the base into the test cage. All replicates were then maintained at 25°C ± 2°C and ~60% RH in the dark for 240 h. Mortality was recorded 3 times daily until 96 h, and thereafter every 24 h until 240 h to allow both LC_50_ calculations at different time points and DEBtox modelling.

Native *Bombus terrestris audax* (11 colonies) were obtained from NV Biobest, Belgium and reared on 50% w/v sucrose supplemented with fresh pollen. Three different Biobest colonies were used separately (i.e. all bees from the same colony) as the source for bees for each of the three replicates that were used for each single chemical and mixture treatment. Test bioassays used groups of three worker bees taken from a single commercially supplied colony within each replicate. Bees were housed in the same flight cages, feeders and conditions as for the honeybee studies.

Overwintered *O*. *bicornis* pupae were stored at 4±1°C, 65±10% RH in constant dark. Cocoons were initially size segregated, with the large pupae expected to correspond mainly to females and smaller pupae as males. To emerge adult bees for experimental studies, an excess of assumed (based on size) male and female pupae were warmed to 25°C for 1–5 day depending on the time of year (shorter later in season). Emergence success was around 80% throughout the testing season (April-June), with storage time having no effect on overall viability.

A detailed study of survival and feeding over the 240 h test duration for female *O*. *bicornis* was carried out. This assessment indicated a cohort effect on the duration of survival. If individuals were maintained directly after hatching, one portion of the population was lost early during husbandry, while a second cohort could be kept alive for 240 h (and beyond). Hence for all experiments with *O*. *bicornis*, after an excess of bees were hatched, the population was initially kept unexposed for 96 h i.e. on feeders containing only sucrose solution. Bees still alive after this time were then subsequently used for the experiments with the expectation that they would survive the 240 h exposure period. For all experiments, 5 males and 5 females were exposed in each test treatment. Individuals were housed separately in the same flight cages as used for the honeybee and bumblebee studies. Feeders comprised smaller syringes containing 5ml of 20% w/v sucrose with yellow false silk petals with a ring of UV paint attached around the feeding hole. Experiments were kept in a controlled temperature glass house at 22 ± 2°C under natural photoperiod. For each replicate, mortality was recorded three times daily during the first 96 h and, thereafter, at 24 h intervals up to 240 h. As clothianidin is subject to photo-degradation with a stated half-life between 1–38 days in water, exposure solutions containing this compound were changed after 5 days as a compromise between excessive disturbance of incubated bees and maintenance of the pesticide in the food source.

### Data analysis for potentiation and mixture experiments and DEBtox modelling

Concentration response analysis for potentiation experiments: All raw data associated with this work is available through Dryad under **doi:**10.5061/dryad.676ng. Each series of tested concentrations of the toxic chemicals in the Potentiation experiments (both those in the absence and presence of the second chemical, See Fig 1 for design) was analysed separately using probit analysis for the data at 48 h, 96 h and 240 h to estimate LC_50_ concentrations in the absence and presence of the second compound. Based on an assumption of no contribution of the second chemical to toxicity, LC_50_s determined for each chemical exposure would be expected to be equivalent. If changes in calculated values are seen when the second chemical is present this would be indicative of an interactive effect corresponding to synergism—lower LC_50_ in the presence of the second chemical, or antagonism—higher LC_50_ in the presence of the second chemical. Significant differences in LC_50_ in each concentration series with added propiconazole or tau-fluvalinate were compared to those from exposures with the second compound using the LC_50_ ratio test [28].

MIXTOX modelling for mixture toxicity experiments: Analysis of the mixture toxicity experiments used both CA and IA as an initial basis for joint effect analysis [12, 13, 15]. By generating CA and IA predictions from the single compound data in each experiment, we were then able to compare observed mixture effects against these predictions using log likelihood testing to assess whether the observed mixture data deviated significantly from the prediction made according to CA and IA from the single chemical only data (approach fully described in [15]). The initial model included parameters relating to the maximum, 50% effect concentrations (EC_50_) and the slope parameter (*b*) of the logistic fits for each of the two chemicals in the mixture. The fit of the CA and IA model was initially assessed against a null model of no joint effect to ensure there was a significant mixture effect. Assuming this was the case, additional functions for synergistic/antagonistic (*a*), concentration-ratio (*b*_*DR*_) and effect level (*b*_*DL*_) deviations were then added in turn to the models to gauge if the extended model significantly improved fit compared to CA or IA using chi-square analysis for the nested models. Fitting the synergism/antagonism model to the data used the parameters generated from the CA and IA model together with an initial value of zero for *a*. If a statistically significant improvement in model fit occurred with the inclusion of *a*, then these parameters were used as starting values for the concentration-ratio and effect-level models with values of zero for *b*_DR_ and two for *b*_DL_. Where significant improvements in the data fit to the model were found, the parameter values can be used to ascertain the nature of the interaction (for full details of the statistical approach see [15]). Mixtox model fits were conducted for the survival data-sets at 48 h, 96 h and 240 h exposure times for all Mixture Toxicity experiments.

### Mixture toxicity modelling for potentiation and mixture toxicity experiments

DEB-theory can integrate different endpoints, such as growth, reproduction and survival within one consistent framework, usually called DEBtox as initially developed by Kooijman and Bedaux [29]. For this analysis, the survival module was used as a stand-alone modelling framework with the complete series of survival measurements for all time points (19 in total) used as the input to the model. The survival DEBtox module model includes a scaled one-compartment model to describe uptake and elimination and a hazard model to describe survival. Four time-independent parameters are derived and used to describe the time course of toxic effect: the Blank Killing Rate as a measure of background mortality (hr^-1^); the No Effect Concentration (NEC) as a toxicological threshold below which no effects occur for any exposure time (mmol/L); the elimination rate (*k*_*e*_), which describes when the equilibrium between internal and external concentration occurs and killing rate (*k*_*k*_) as the toxic potency once the NEC is exceeded (mmol/L^-1^ d^-1^).

This survival modelling framework of DEBtox was initially developed for single compound data, but has been extended to analyse data from Mixture Toxicity experiments by Baas et al. [19]. For the adaptation, an additional interaction parameter is incorporated into the extended model. When a model fit indicates that the interactions parameter does not improve the model fit, then the mixture is taken to be additive within the DEBtox framework and survival probability of the mixture are the product of those of the individual compounds in the mixture. If an interaction is found, then any additional parameters included in the model will provide a significantly improved fit of the model to observed effects in time over the dataset. This application of DEBtox for mixtures to analyse survival effects in time, thus, gives valuable insights in the overall nature of synergistic or antagonistic effects and, through the parameter values of the model, can give toxicokinetic and toxicodynamic insights relevant to the case.

## Results

Parameters derived from model fits for the probit analysis for the Potentiating experiments, MIXTOX model fits for the Mixture Toxicity experiments and DEBtox model fits for both experiment types are given in Tables 2, 3 and 4 respectively.

**Table 2:** LC_50_ values as concentrations in sucrose solution (μg/L) calculated from probit models fits and ratio of values between exposure in the presence of low and high concentration of the potentiating chemical against exposure in the absence of the second chemical for the survival data at 48h, 96 h and 240 h exposure times for the different potentiation experiments conducted for *Apis mellifera*, *Bombus terrestris* and separately for ♂ and ♀ *Osmia bicornis*.

	*48 h LC*_*50*_ *(95% CIs)* μg/L	*Ratio +/-*	*96 h LC*_*50*_ *(95% CIs)* μg/L	*Ratio +/-*	*240 h LC*_*50*_ *(95% CIS)* μg/L	*Ratio +/-*
***A*. *mellifera***						
Dimethoate only	2.67 (2.26–3.08)		1.16 (0.97–1.35)		0.624 (0.525–0.723)	
Dimethoate low propiconazole	3.05 (2.57–3.54)	0.88	1.55 (1.31–1.80)	0.75	0.508 (0.407–0.609)	1.23
Dimethoate high propiconazole	2.86 (2.40–3.32)	0.93	1.35 (1.13–1.58)	0.86	0.504 (0.433–0.647)	1.24
***A*. *mellifera***						
Clothianidin only	0.22 (0.17–0.27)		0.128 (0.11–0.15)		0.07 (0.057–0.082)	
Clothianidin low propiconazole	0.190 (0.161–0.219)	1.16	0.143 (0.12–0.166)	0.9	0.054 (0.044–0.064)	1.3
Clothianidin high propiconazole	0.21 (0.158–0.262)	1.05	0.122 (0.101–0.143)	1.05	0.053 (0.042–0.064)	1.32
***A*. *mellifera***						
Clothianidin only	0.20 (0.167–0.233)		0.16 (0.131–0.189)		0.074 (0.057–0.091)	
Clothianidin low tau-fluvalinate	0.17 (0.141–0.199)	1.18	0.125 (0.102–0.148)	1.28	0.066 (0.053–0.080)	1.12
Clothianidin high tau-fluvalinate	0.152 (0.126–0.178)	1.32	0.129 (0.104–0.153)	0.97	0.075 (0.052–0.098)	0.88
***B*. *terrestris***						
Clothianidin only	0.027 (0.019–0.035)		0.018 (0.013–0.023)		0.016 (0.011–0.021)	
Clothianidin high propiconazole	0.015 (0.011–0.019)	1.8	0.013 (0.009–0.016)	1.38	0.012 (0.009–0.016)	1.33
***B*. *terrestris***						
Clothianidin only	0.027 (0.019–0.035)		0.018 (0.013–0.023)		0.016 (0.011–0.021)	
Clothianidin low tau-fluvalinate	0.02 (0.016–0.024)	1.35	0.018 (0.014–0.021)	1	0.015 (0.012–0.019)	1.07
Clothianidin high tau-fluvalinate	0.018 (0.014–0.022)	1.5	0.012 (0.007–0.017)	1.5	0.007 (0.003–0.011)	2.29
**♂ *O*. *bicornis***						
Dimethoate only	4.65 (2.34–6.96)		0.6 (0.31–0.89)		0.6 (0.31–0.89)*	
Dimethoate low propiconazole	2.62 (-)	1.77	0.80 (-)	0.75	0.435 (0.255–0.615)	1.38
Dimethoate high propiconazole	3.98 (2.74–5.23)	1.17	-	-	0.368 (0.236–0.501)	1.63
**♀ *O*. *bicornis***						
Dimethoate only	3.63 (2.4–4.85)		1.011 (0.56–1.46)		-	-
Dimethoate low propiconazole	2.25 (-)	1.61	0.68 (0.42–0.94)	1.51	-	-
Dimethoate high propiconazole	2.25 (-)	1.61	1.024 (-)	-	-	-
**♂ *O*. *bicornis***						
Clothianidin only	0.197 (0.119–0.275)		0.172 (0.101–0.242)		0.058 (0.017–0.098)	
Clothianidin low propiconazole	0.090 (0.051–0.130)	2.18	0.084 (0.042–0.126)	2.05	0.056 (0.021–0.091)	1.04
Clothianidin high propiconazole	0.063 (0.037–0.089)	3.11	0.050 (0.025–0.076)	1.68	0.025 (0.007–0.044)	2.24
**♀ *O*. *bicornis***						
Clothianidin only females	0.058 (0.038–0.078)		0.046 (0.024–0.068)		0.036 (0.021–0.050)	
Clothianidin low propiconazole	0.051 (-)	1.14	0.042 (0.025–0.059)	1.1	0.031 (0.016–0.046)	1.16
Clothianidin high propiconazole	0.048 (0.030–0.067)	1.21	0.048 (0.030–0.067)	0.96	0.036 (0.021–0.050)	0.86

* Value calculated for 168 h.

**Table 3:** Parameter values (EC_50_, *b* = logistic slope parameter, *a* synergistic/antagonistic deviation, *B*_*DR*_ dose ratio deviation *B*_*DL*_ *dose level deviation*) for MIXTOX models fits for the two chemicals (Chemical 1 relates to the first listed chemical, Chemical 2 to the second listed chemical) used in binary mixtures survival data at selected time-points for mixture toxicity experiments conducted for *A*. *mellifera*, *B*. *terrestris* and *O*. *bicornis*.

	Model	Exposure time (h)	Model r^2^	Max	Chem 1 *b*	Chem 1 EC_50_ μg/L	Chem 2 *b*	Chem 2 EC_50_ μg/L	*a*	*b*_*DR*_	*b*_*DL*_
***A*. *mellifera***											
Clothianidin & dimethoate	CA	96	0.809	0.945	2.44	0.087	9.57	1.35	2.47***		
Clothianidin & dimethoate	CA	240	0.728	0.913	2.36	0.052	3.71	0.615	3.2***		
Clothianidin & dimethoate	IA	96	0.788	0.98	2.19	0.082	8.29	1.3	1.59***		
Clothianidin & dimethoate	IA	240	0.712	0.98	1.87	0.044	3.02	0.569	3.85***		
***A*. *mellifera***											
Clothianidin & Cadmium	CA	96	0.830	0.952	5.87	0.194	26.3	8.69			
Clothianidin & Cadmium	CA	240	0.827	0.971	1.49	0.054	2.98	9.17	5.77***		
Clothianidin & Cadmium	IA	96	0.799	0.98	3.65	0.115	2.41	12.4			
Clothianidin & Cadmium	IA	240	0.805	0.98	1.97	0.056	3	9.263			
***A*. *mellifera***											
Cadmium & Arsenic	CA	96	0.817	0.98	1.61	22.3	4.30	11.7			
Cadmium & Arsenic	CA	240	0.689	0.98	0.97	16.9	4.47	4.97			
Cadmium & Arsenic	IA	96	0.825	0.98	2.62	12.6	3.7	10.6			
Cadmium & Arsenic	IA	240	0.695	0.98	1.12	13.1	4.37	4.93			
***B*. *terrestris***											
Clothianidin & dimethoate	CA	96	0.729	0.914	6.25	0.021	5.27	1.47	1.74**		
Clothianidin & dimethoate	CA	240	0.752	0.741	19.1	0.014	5.63	0.355	4.78***		
Clothianidin & dimethoate	IA	96	0.720	0.89	5.78	0.022	18.40	1.35			
Clothianidin & dimethoate	IA	240	0.638	0.98	4.26	0.013	3.27	0.261	0.436***		-16.9*
**♀ *O*. *bicornis***											
Clothianidin & dimethoate	CA	96	0.902	0.980	4.39	0.845	0.476	0.486			
Clothianidin & dimethoate	CA	240	-	-	-	-	-	-			
Clothianidin & dimethoate	IA^1^	96	0.807	0.95	2.91	0.389	0.206	5.85	584*		
Clothianidin & dimethoate	IA^1^	240	0.973	0.98	9.9	0.06	1.47	0.051			
**♂ *O*. *bicornis***											
Clothianidin & dimethoate	CA	96	0.847	0.782	2.798	0.420	0.684	0.999	59.4*		
Clothianidin & dimethoate	CA	240	-	-	-	-	-	-			
Clothianidin & dimethoate	IA^1^	96	0.807	0.95	2.91	0.389	0.206	5.85	584*		
Clothianidin & dimethoate	IA^1^	240	0.973	0.98	9.9	0.06	1.47	0.051			

* p<0.05,

** p<0.01,

*** p<0.001.

**Table 4:** Parameter values (values in brackets are standard deviations) for DEBtox models fit for the potentiation and mixture toxicity experiments conducted in *A*. *mellifera*, *B*. *terrestris* and *O*. *bicornis*.

	Blank Killing rate (hr^-1^)	*NEC* *(mg/L)*	*Killing rate* *(mg/L)^-1^hr^-1^)*	*Elimination rate* *(h^-1^)*	*interaction*?
***A*. *mellifera***					
dimethoate only	1.8 x 10^−4^ (0.7 x 10^−4^)	040 (003)	0057 (011)	001 (0002)	
dimethoate low propiconazole	3.1 x 10^−4^ (1 x 10^−4^)	030 (004)	0062 (0014)	0007 (0002)	No
dimethoate high propiconazole	3.1 x 10^−4^ (1 x 10^−4^)	035 (004)	0046 (0009)	0011 (0002)	
***A*. *mellifera***					
clothianidin only	2.6 x 10^−4^ (1.1 x 10^−4^)	002 (0004)	0093 (0002)	0041 (002)	
clothianidin low propiconazole	3.1 x 10^−4^ (1.2 x 10^−4^)	0015 (0005)	0097 (002)	0028 (0011)	No
clothianidin high propiconazole	4.1 x 10^−4^ (1.4 x 10^−4^)	0017 (0004)	012 (002)	0031 (0011)	
***A*. *mellifera***					
clothianidin only	9 x 10^−4^ (2 x 10^−4^)	004 fixed^1^	0093 (0012)	1.06 (1.40)	
clothianidin low tau-fluvalinate	6.8 x 10^−4^ (1.6 x 10^−4^)	0038 (0005)^1^	012 (002)	084 (083)	No
clothianidin high tau-fluvalinate	5.0 x 10^−4^ (1.0 x 10^−4^)	0017 (0004)^1^	012 (002)	025 fixed	
***A*. *mellifera***					
clothianidin	2.8 x 10^−4^ (1.4 x 10^−4^)	0025 (0003)	017 (003)	013 (007)	No^2^
dimethoate	4.5 x 10^−4^ (1.4 x 10^−4^)	030 (011)	0030 (001)	0008 (0004)	
***A*. *mellifera***					
clothianidin only	Reliable parameter estimates not possible, but expected NEC 0.04 mg/L				
clothianidin with Cd	No parameter estimates possible				
***A*. *mellifera***					
As only	8.4 x 10^−4^ (0.2 x 10^−4^)	4.4 (0.77)	3.8 x 10^−3^ (0.9 x 10^−3^)	0.016 (0.004)	No
As low Cd	6.2 x 10^−4^ (2.1 x 10^−4^)	2.96 (1.10)	3.4 x 10^−3^ (0.8 x 10^−3^)	0.017 (0.007)	
As high Cd	5.9 x 10^−4^ (2 x 10^−4^)	3.75 (0.92)	3.4 x 10^−3^ (0.8 x 10^−3^)	0.019 (0.0061)	
***B*. *terrestris***					
clothianidin only	5.6 x 10^−4^ (1.8 x 10^−4^)	23.9 (1.1)	0.0061 (0.0030)	0.30 (0.11)*	Possible
clothianidin high propiconazole	6.3 x 10^−4^ (2.0 x 10^−4^)	10.9 (8.7)	0.0060 (0.0034)	0.19 (0.12)*	synergism
***B*. *terrestris***					
clothianidin only	1.30 x 10^−4^	46.7	0.12	0.004	No
clothianidin low tau-fluvalinate	4.00 x 10^−4^	21	0.01	1	
clothianidin high tau-fluvalinate	Parameters not calculated due to presence of effect from propconazole				
***B*. *terrestris***					
Clothianidin	10 x 10^−4^ (3.0 x 10^−4^)	23.1 (1.6)	0.0071 (0.0035)	0.47 (0.2)*	Possible Antagonism
Dimethoate	1.2 x 10^−3^ (0.0004)	0.097 (0.077)	0.35 (0.30)*	1.7 x 10^−3^ (1.5 x 10^−3^)	Antagonism
**♂ *O*. *bicornis***					
dimethoate only	1.2 x 10^−3^ (6.8 x 10^−4^)	0.32 (0.13)	0.030 (0.016)	0.027 (0.018)	
dimethoate low propiconazole	6.7 x 10^−4^ (4.7 x 10^−4^)	0.26 (0.09)	0.27 (0.16)*	0.0085 (0.004)	No
dimethoate high propiconazole	7 x 10^−4^ *	0.25 (0.09)	0.10 *	0.014 (0.005)	
**♀ *O*. *bicornis***					
dimethoate only					
dimethoate low propiconazole	No parameter estimates possible				No
dimethoate high propiconazole					
**♂ *O*. *bicornis***					
clothianidin only					
clothianidin low propiconazole	No parameter estimates possible				No
clothianidin high propiconazole					
**♀ *O*. *bicornis***					
clothianidin only					
clothianidin low propiconazole	No parameter estimates possible				
clothianidin high propiconazole					
**♂ *O*. *bicornis***					
Clothianidin	No parameter estimates possible				No
Dimethoate					
**♀ *O*. *bicornis***					
Clothianidin	7.0 x 10^−4^ *	0.26 (0.13)	0.19 (0.12)	0.005 (0.004)	No
Dimethoate	7.0 x 10^−4^ *	0.04 (0.21)	0.25 (1.4)	0.02 (0.10)	

^1^ NEC of all three experiments behaves identical: first min at 0.02, second at 0.04, third at 0.06; Indicated values give the best fit, however, the alternative values may be equally valid

^2^ p = 0.05

* parameter difficult to estimate due to asymptotic behaviour

### Potentiation experiments

#### Dimethoate + propiconazole

*Apis mellifera*: Dimethoate 48 h, 96 h and 240 h LC_50_ values decreased with time in each exposure series (Table 2). Lethal toxicity values conducted in the presence of propiconazole were not significantly different from that for clothianidin alone across all time points (LC_50_ ratio test p>0.05 in all cases) indicating no potentiation or alleviation of dimethoate toxicity by propiconazole (Table 1). DEBtox model NECs, elimination rates and killing rates for dimethoate were almost identical for each propiconazole exposure series (Table 4). Again this suggests no interactions between the chemicals.

*Osmia bicornis*: *Osmia bicornis* probit fits were weaker than those for *A*. *mellifera* as indicated by the larger 95% confidence intervals for the calculated LC_50_s (Table 2). A difference of no more than a factor of 2 was observed between LC_50_ values for dimethoate, either alone, or in the presence of high or low propiconazole concentrations at all times (< factor of 2). However, this potentiation was not significant for any comparison to the dimethoate only value for any time point (LC_50_ ratio test p>0.05 in all cases). This was the case for both male and female bees. At each time interval, the lowest LC_50_ value was most frequently found in the exposures with the highest propiconazole concentration, especially for male bees. This observation points to a small degree of potentiation. DEBtox fits for male *Osmia bicornis* showed lower NECs in the presence of propiconazole (Table 4).

Estimated elimination rate was also reduced 2 and 3 fold in the presence of low and high propiconazole concentrations, although confidence intervals of the parameter estimates overlapped for different exposure series. Killing rate increased in the presence of propiconazole, although estimation of this parameter was difficult due to asymptotic behaviour. These differences, namely lower NECs, elimination rates and killing rates, suggest a possible marginal effect of propiconazole on dimethoate toxicity in male bees. However, in all cases the differences in parameter values are small and the overlap of confidence intervals indicates that differences are not significant. Unequivocal DEBtox fits could not be derived for female bees, with numerous parameter combinations giving nearly equal fits. The majority of parameters suggested similar values in ± propiconazole treatments, implying no potentiation in females.

#### Clothianidin ± propiconazole

*Apis mellifera*: Clothianidin LC_50_s reduced considerably when exposure was extended from 96 h to 240 h (Table 2). The lowest LC_50_s were found when clothianidin exposure took place in the presence of the high propiconazole concentration at 96 h and 240 h, but not at 48 h when the lowest value was for exposure in the presence of low propiconazole (Fig 1a). However, these differences were not significant (LC_50_ ratio test p>0.05 in all cases). DEBtox parameter values for clothianidin toxicity were slightly modified by the presence of propiconazole (Table 4). While calculated NECs were almost identical, the modelled elimination rates were lower and killing rates higher for exposures conducted in the presences of propiconazole. This may indicate a limited modifying effect of propiconazole on clothianidin toxicokinetics and toxicodynamics.

**Fig 2:**
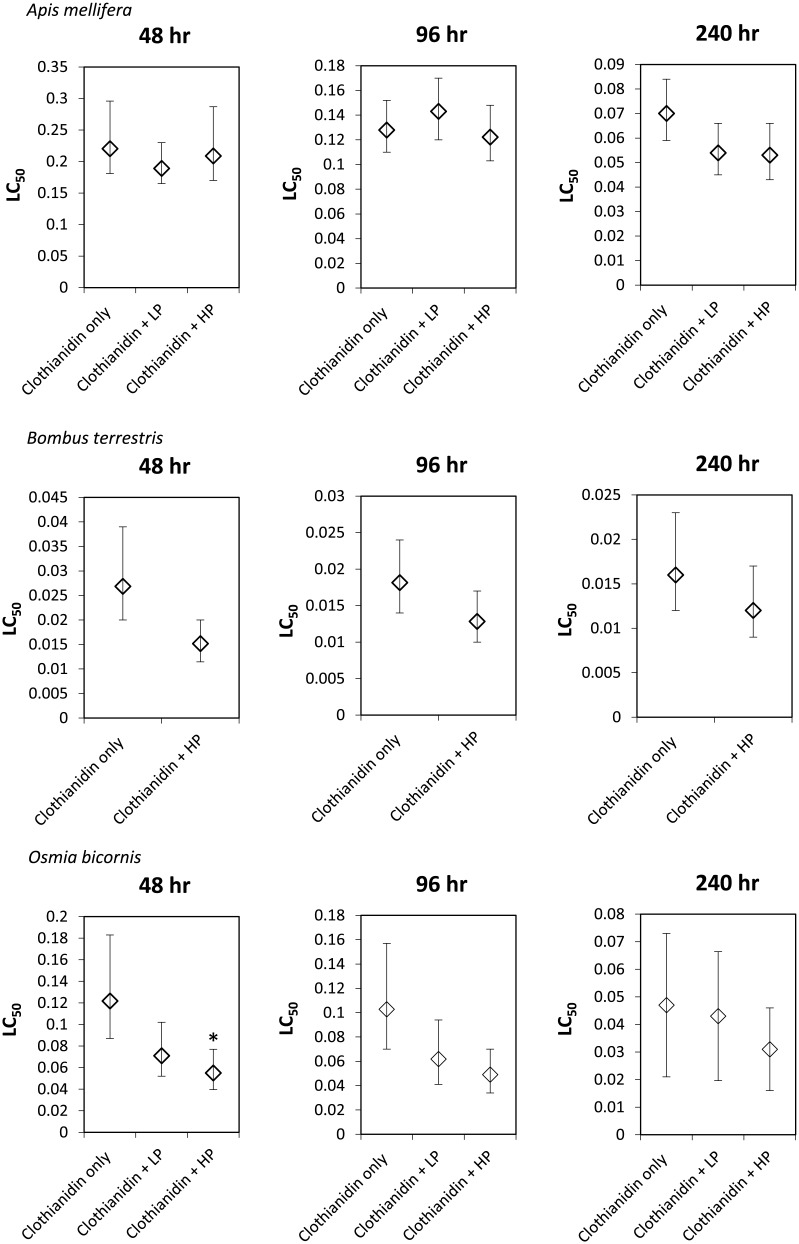
LC_50_ ± 95% confidence intervals and statistical significance of treatments with propiconazole compared to the propiconazole only series (LC_50_ ratio test * = p<0.05)for *Apis mellifera* (top 3 panels), *Bombus terrestris* (middle 3 panels), and the combined data-set of ♂ and ♀ *Osmia bicornis* (bottom 3 panels) exposed to clothianidin in the presence of no, low or high concentrations of propiconazole at exposure times of 48 h (Panel 1 of 3), 96 h (Panel 2 of 3) and 240 h (Panel 3 of 3).

*Bombus terrestris*: Clothianidin LC_50_s decreased over time, falling approximately 2 fold on extending the exposure from 48 h to 240 h of the (Table 2). A slight synergistic effect of propiconazole was indicated as clothianidin LC_50_s were decreased by 1.5 to 2 fold in the presence of high propiconazole concentrations compared to that without co-exposure (Fig 2b). These differences in LC_50_s were not significant for any time point (LC_50_ ratio test p>0.05 in all cases). DEBtox fits indicated that propiconazole addition led to changes in clothianidin NEC and elimination rates. Change of the NEC value indicates a higher sensitivity in the presence of the fungicide in a manner consistent with a potentiating effect of a factor of 2.

*Osmia bicornis*: Clothianidin LC_50_s consistently decreased with time in all exposure series. For male bees, there was a 3 fold reduction in LC_50_ in the high propiconazole treatment compared to the value without propiconazole (Fig 2c, Table 2). This reduction was significant (LC_50_ ratio test p = 0.03) for the high propiconazole treatment compared to clothianidin only at 96 h and close to significant (LC_50_ ratio test p = 0.08 in all cases) for the same comparison at 48 h. This points to a potentiating effect of the fungicide on clothianidin toxicity for male *O*. *bicornis*. DEBtox models for male and female bees in exposure ± propiconazole identified a small change in the NEC, elimination rate and killing rate parameters, which are not reported here in detail because they are based on weak model fits. These models for males and females also identified no interaction between the two chemicals. It is, however, possible that the failure to identify such response patterns may be a consequence of the weakness of the model fits for the separate male and female datasets.

#### Clothianidin ± tau-fluvalinate

*Apis mellifera*: Clothianidin LC_50_ values were lower in the presence of the high tau-fluvalinate concentration at 48 h, but not at later time-points (Table 2). This suggests no consistent modifying effect of tau-fluvalinate on clothianidin toxicity. Within DEBtox fits, the NEC was a factor of 2 lower in the test conducted in the presence of high tau-fluvalinate concentrations than that in exposures with tau-fluvalinate only (Table 4). All three clothianidin exposure series could be fitted with different sets of DEBtox parameter values that each gave comparable fits. NECs of 0.02, 0.04 and 0.06 mg/L gave a similar log likelihood estimated goodness of fit. In the absence of tau-fluvalinate and at in low concentrations series, a model with a NEC of approximately 0.04 mg/L gave best fit. In the presence of high tau-fluvalinate, a model with a NEC of 0.02 mg/L for clothianidin gave the best fit. The elimination rate was not strongly fixed by the data but reduced approximately 4-fold between the low and high concentration tau-fluvalinate tests. Although this difference was not significant, changes for both values in the presence of tau-fluvalinate tentatively suggest a possible small magnitude effect of high concentrations on clothianidin sensitivity and handling. However, such changes if present evidently lead to only marginal effects on observed toxicity.

*Bombus terrestris*: Against a background of decreasing clothianidin LC_50_ with time, an up to 1.5 fold potentiating effect of high level tau-fluvalinate co-exposure was seen across all time-points (Table 2). However, observations of an effect on survival at high level tau-fluvalinate means that changes in survival observed are likely to result from tau-fluvalinate toxicity rather than any potentiation. DEBtox model fits for clothianidin effects on survival gave a range of possible parameter values. The model fits were similar in the absence and presence of tau-fluvalinate and were not enhanced by inclusion of an interaction parameter (Table 4). This finding suggests independent and additive effects for the two chemicals rather than any interactive toxicity to give greater than or less than additive toxicity.

### Mixture toxicity experiments

#### Clothianidin + dimethoate

*Apis mellifera*: Both CA and IA provided a very significantly improved fit compared to the null model of no joint effects for all time-points. Addition of the S/A (synergism / antagonism) parameter significantly improved both CA and IA fits for the mixture (Table 3). The value of *a* in the extended model was >1 indicating predominantly antagonism. Addition of further parameters to the model did not significantly improve the model fit. The DEBtox model of the effects data showed a good overall fit, which was further improved by the addition of a parameter allowing for antagonism. The improvement was at the boundary of significance (p = 0.05).

*Bombus terrestris*: MIXTOX models for all time points indicated a high significance of both the CA and IA models against the hypothesis of no joint effects. CA (96 h r^2^ = 0.73, 240 h r^2^ = 0.75) marginally better described the observed data than IA (96 h r^2^ = 0.72, 240 h r^2^ = 0.64). Addition of the S/A parameter to both the CA and IA significantly improved fits to the observed data at 240 h and also at 96 h for CA, while the IA S/A model at this exposure time was close to significant at p = 0.07. The value for *a* was >1 for all significant CA and IA S/A models indicating antagonism in the mixture. Addition of the b_DL_ parameter further significantly improved the model fit at 240 h. The value of b_DL_ was <1, suggesting greatest antagonism at high exposure levels. Within DEBtox models, NEC values and killing rates for the mixture treatments were comparable (Table 4). Observed survival was generally higher than predicted from the combined single chemicals models for later exposure times in the different mixture treatments. This provides evidence of a possible antagonistic interaction for this mixture.

*Osmia bicornis*: Both CA and IA gave a significant improved fit compared to the alternative hypothesis of no joint effect. For males at 96 h, including the *a* parameter significantly improved the fit of both reference models to the observed effects. The value of *a* was positive, indicating antagonism. Inclusion of b_DL_ and b_DR_ did not further significantly improved the model fits. For female *O*. *bicornis*, the models including interaction parameters improved the 96 h IA model fit, with a positive value for *a* indicating antagonism for effects at this time point. Joint effect of these two chemicals were additive (or could not be reliably fitted) at 240 h. DEBtox models fits for female *O*. *bicornis* (n.b. an unequivocal model fit could not be obtained for males) were not improved by inclusion of an interaction parameter indicating mainly additive effects across the full exposure time course.

#### Arsenic & cadmium

*Apis mellifera*: Both the CA and IA fits were highly significant against a model of no joint effect. Since 50% mortality was only approached in the top Cd treatment at 240 hours, model fits were weaker for previous time points. IA fitted the data slightly better than CA, although this difference was small meaning that conclusions on the most appropriate model can only be preliminary. Inclusion of interaction parameters failed to significantly improve the fit of either reference models for all time-points (Table 3). This suggests an additive joint effect. DEBtox fits for each metal indicated no effect on parameter values resulting from co-exposure, with the interaction parameter also not significantly improving the model fit (Table 4). Again this suggests non-interactive additivity.

#### Clothianidin & cadmium

*Apis mellifera*: Effects of clothianidin in this mixture only became apparent after 192 hrs exposure and Cd effects were only observed at the highest dose (8.32 mg/L) after 96 hours. Hence, interpretation of joint effects is limited to later time-points. MIXTOX model fits for the 96 h and 240 h survival data were each highly significant against the alternative model of no joint effect. The S/A model for CA identified a significant interaction at 240 h, but not 96 h (Table 3). The value for *a* was >1 suggesting antagonism. Addition of parameters to the IA models failed to significantly improve fits for any time-point, indicating an additive effect according to IA assumptions. Since the dosing was such that significant effects on survival were only observed after 192 h, only limited data was available for interpretation of joint effect using DEBtox. Initial analysis suggested that survival following exposures to clothianidin were significantly higher in the presence of Cd, suggesting an antagonistic effect of Cd on clothianidin toxicity. However, this observation is derived from only a weak model fit. Specific inclusion of an interaction term did not significantly (p > 0.05) improve the overall model fit, suggesting non-interactive additivity for this mixture across the full time course.

## Discussion

The capacity to identify additive, synergistic or antagonistic responses to chemical mixture exposures can contribute to the development of more comprehensive hazard assessment of complex chemical exposures in bee species. Identifying the mixtures that are additive can support hazard assessment using established prediction based approaches based on CA and IA and can also allow interactive (synergistic, antagonistic) chemical combinations to be identified. By comparing observed response patterns for any given mixture across different species, it is possible to establish whether the patterns of joint or interactive effects (additive, synergism, antagonism) are species specific or common. Cases of greatest concern are those mixtures that elicit synergistic toxicity effects. Concern arises because risk assessments that use toxicological data for single chemicals and predictions using established models may fail to provide adequate protection in such cases. Where synergistic interactions are consistent between species, this will be associated with elevated risk not just for a single species but for all species that together deliver pollination services [30]. As such, synergistic effects represent a currently unassessed risk to ecosystem service provision, as specifically recognised by Sanchez-Bayo and Goka [11].

Tests for the binary mixtures studies in *A*. *mellifera* and for the dimethoate and propiconazole mixture in *B*. *terrestris* and *O*. *bicornis* identified a number of combinations showing non-interactive additive toxicity (see Table 5 for summary of main response patterns from all experiments). Hence for many cases, current models for mixture toxicity may be able to adequately predict effects. In other cases mixture interactions were found. Two Potentiating experiments with *A*. *mellifera*, namely dimethoate ± propiconazole and clothianidin ± tau-fluvalinate, and one Mixture Toxicity experiment, with As and Cd, showed independent joint effects. For the Potentiation mixture with dimethoate ± propiconazole that same non-interactive pattern of joint effects was also seen in the study with *B*. *terrestris* and *O*. *bicornis*, suggesting that the absence of any interaction was taxonomically conserved. DEBtox model fits that analysed all data across the time-courses for all combinations, also results in fits for these mixtures that were not improved by an interaction parameter. The two reference joint effect models for similarly and dissimilarly acting mixtures of CA and IA, or non-interaction DEBtox models, therefore, offer a simple approach to conduct joint hazard assessment for these mixtures in bees.

**Table 5:** Summary of the nature of interactions identified in the joint effects of binary combinations of chemicals in potentiation and mixture toxicity experiment conducted with three bee species.

	96 h CA	96 h IA	DEBtox
***Apis mellifera***			
Dimethoate + propiconazole	No potentiation	No potentiation	No potentiation
Clothianidin + propiconazole	**Very slight potentiation**	**Very slight potentiation**	No potentiation
Clothianidin + tau-fluvalinate	No potentiation	No potentiation	No potentiation
Dimethoate + clothanidin	**Antagonism**	**Antagonism**	Additive
Clothianidin + Cd	**Slight antagonism**	Additive	Additive
Cd + As	Additive	Additive	Additive
***Osmia bicornis***			
Clothianidin + propiconazole	**Moderate potentiation**	**Moderate potentiation**	No interaction
Clothianidin + tau-fluvalinate	No potentiation	No potentiation	No potentiation
Dimethoate + clothanidin	**Antagonism ♂ only**	**Antagonism ♂ only**	Additive
***Bombus terrestris***			
Dimethoate + propiconazole	No potentiation	No potentiation	No potentiation
Clothianidin + propiconazole	**Slight potentiation**	**Slight potentiation**	No potentiation
Dimethoate + clothanidin	**Antagonism**	**Antagonism**	**Possible antagonism**

The Mixture Toxicity experiment with clothianidin and Cd found slight antagonism compared to CA predictions at 240 h exposure, but not at 96 h or compared to IA model predictions. DEBtox analysis also suggested the presence of a possible interaction, although based on only a weak model fit. Hence, interactions seen for these two chemicals are of only small magnitude and appear both time-point dependent and relevant only to CA predictions. Previous studies have identified some effects of trace metal exposure on organic chemical toxicokinetics. Broerse et al. [31] found that Cd increases the hydroxylation rate of a polyaromatic hydrocarbon, pyrene, but slowed down its further metabolization in the Collembolan *Folsomia candida*, resulting in a prolonged half-life of first phase hyrodoxlated pyrene metabolites. An interactive effect on chlorpyrifos insecticide and nickel metal toxicity was found in the ground beetle *Pterostichus oblongopunctatus*, however chlorpyrifos reduced nickel accumulation [32]. In contrast, other chlorpyrifos and nickel toxicity studies on earthworms [33] and marine mussels [34] yielded no interaction effects. Since interactions have been found in the two arthropod studies, but not in other taxa, interactions may be more common within the Arthropod phylum than in other groups.

For the remaining two binary mixtures tested, namely the Potentiating experiment with clothianidin ± propiconazole and the Mixture Toxicity experiment with clothianidin and dimethoate, response patterns that were fully consistent with additivity were observed. The highest difference found for the change in LC_50_ in a mixture exposure was a factor of 3 fold, although the majority of differences were smaller than this. These interactive response patterns were largely consistent across species, models and analysis methods. This indicates that, for these two mixtures, more complex models that account for non-independent and non-additive joint effects may be required for valid hazard prediction.

For the clothianidin and propiconazole Potentiation mixtures in *A*. *mellifera* only small magnitude non-significant potentiation was found on co-exposure with higher concentration of propiconazole (Fig 2a). The pattern of potentiation pattern was, however, more evident in the propiconazole exposures conducted for *B*. *terrestris* (Fig 2b) and *O*. *bicornis* (Fig 2c), reaching a maximum 3-fold statistically significant difference of 96 h LC_50_ values for *O*. *bicornis* exposed to clothianidin in the presence of high propiconazole compared to without (Fig 2b). Further, for male *O*. *bicornis*, potentiation was also seen in the low propiconazole series (Fig 2c).

Previous studies of combined exposure under a potentiating experimental design have identified synergetic interactions between neonicotinoids and known P450 inhibitors including the sterol biosynthesis inhibiting fungicide propiconazole [8]. Synergism seen was greater for cyano-substituted neonicotinoids (e.g. thiacloprid and acetamiprid) as compared to the nitro-substituted neonicotinoids such as imidacloprid. Given that clothianidin is a nitro-substituted compound, the relatively small-scale, although largely temporally and taxonomically conserved, synergisms seen here are consistent with a small modifying effect of this class of fungicide on the toxicity of the neonicotinoid class. In a study that assessed the toxicity of the pyrethroid tau-fluvalinate in the presence of a number of sterol biosynthesis inhibiting fungicides, potentiations of toxicity ranging up to 100 fold for prochloraz were also found, although most were in the 2–5 fold range [6]. These combined results across different studies, suggest that similar synergism may occur both for neonicotinoids and also pyrethroids when exposure occurs in the presence of a sterol biosynthesis inhibiting fungicides. Hence, further work on the potential for neonicotinoid and other insecticides synergism by this fungicide class is needed to assess the range of interactions between different insecticide and fungicide combinations.

The potential for interactions between insecticides and sterol inhibiting fungicides can be mechanistically attributed to the inhibition of key first phase xenobiotic metabolising enzymes such as those of the cytochrome P450 system, by the fungicide [8, 35]. Such inhibition can prevent first phase detoxification of the insecticide, leading to elevated target site exposure of the active chemical and as a result greater realised toxicity. Tau-fluvalinate is known to be highly metabolised by honeybees and this extensive detoxification may be a primary cause of the relatively low toxicity of this pyrethroid for *A*. *mellifera* [36]. Hence cytochrome P450 inhibition by sterol inhibiting fungicides should have a profound synergistic effect. This expectation is consistent with the previous study of Johnson et al. [6] as indeed the authors identify. Like tau-fluvalinate, the neonicotinoid clothianidin is also subject to first phase metabolism by cytochrome P450s. Accordingly inhibition of this class of enzymes through propiconazole co-exposure has the potential to cause synergisms, as seen here in agreement with the observations of Iwasa et al. [8]. Notably, the synergism seen in this work were of relatively small scales. This small magnitude can be could be for two possible reasons: i) clothianidin belongs to the nitro-substituted group of neonicotinoids, which do not seem to be affected much by the fungicide as shown by Iwasa et al. [8]; and, ii) the use of oral exposure in the current study, as compared to topical application by Iwasa et al. [8], which may result in a lower realised internal concentration. However, despite differences in scale, as trends towards synergism were commonly observed (Table 5), this interactive mechanism appears a common trait for many bee species. This consistency of observed synergism indicates that such interactions may need to be considered in ongoing assessments of the hazard of insecticide and sterol biosynthesis inhibiting fungicide mixtures for hymenoptera species [11].

**Fig 3:**
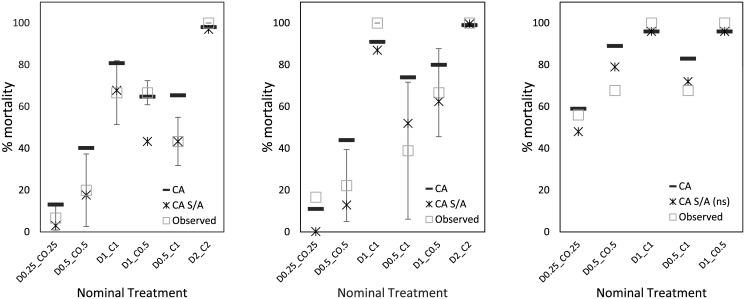
Predicted mixture hazard based on two parameterised mixture fits for concentration addition (CA) and concentration addition with synergistic/antagonistic (CA S/A) models for survival effects at 96 h for *Apis mellifera*, *Bombus terrestris* and *Osmia bicornis* exposed to a range of single chemical and binary mixtures of clothianidin and dimethoate and observed survival effects based mean ± standard deviation.

In all three species tested, the clothianidin and dimethoate mixture showed indication of an antagonistic response pattern (Table 3, Fig 3). This pattern meant that in clothianidin and dimethoate mixtures, observed effects on survival were less that predictions for CA and IA models, as illustrated for CA fits for *A*. *mellifera* (Fig 3a), *B*. *terrestris* (Fig 3b) and *O*. *bicornis* (Fig 3c). While both clothianidin and dimethoate are known to be metabolised by the insect cytochrome P450 systems, the impact of this metabolism is different for the two insecticides. Dimethoate is metabolically activated, increasing in toxicity following the first phase metabolism to dimethoxon, while clothianidin is metabolically detoxified. Hence potential exists for enzymatic competition to cause interactive toxicity based on different rates of transformation and substrate affinity. A further consideration is that although supplied at similar effect levels in mixture treatments (both at 0.25 toxic units, both at 0.5 toxic units etc.), the actual concentration of dimethoate in such equitoxic mixtures will exceed those of clothianidin by at least an order of magnitude due to the higher potency of the neonicotinoid. If the higher concentrations of dimethoate present induce greater cytochrome P450 isozyme expression and clothianidin is a favoured substrate for the metabolising enzymes, then the presence of dimethoate may result in higher neonicotinoid metabolism leading to a reduced toxicity for the mixture. This would be consistent with an antagonistic joint effect as observed across tested species (Table 2).

The co-application of pesticides is a common phenomenon in agricultural systems both through deliberate co-exposures such as tank mixes and sequential application. Modern farming practices have progressively increased the number of active ingredients that are applied to crops during the growing season. For example, in the UK there has been an approximate 50% rise in the average number of active ingredients applied to arable crops over the past 15 years from approximately 11 unique chemistries applied in 2000 to 17 in 2015 [37]. The average is for 2.6 active ingredients to be included in each spray round which may include mixture of insecticides and fungicide or fungicides and herbicide, but rarely combined insecticide mixtures, although these may be used in close sequence. Hence with a variety of compound groups in widespread use, scenarios involving exposure to insecticide and fungicides together and also in the presence of other environmental contaminants are highly relevant scenarios for chemical risk assessment. As honeybees, bumblebees and solitary bees all forage widely in the landscape, they will inevitably be exposed to mixtures corresponding to the types used in this study.

Current schemes to assess the hazards and risks of chemical exposure to bee species take a single chemical approach, with data on the exposure and hazard of each chemical being collated and used independently for risk assessment of each compound; this approach is recognised as rather over-simplistic [38]. While the existence of mixture effects in nature is well known and may be considered for specific scenarios, it is not a regulatory requirement. This is despite the fact that the need for improved understanding of mixture effects has been widely recognised [1, 30, 39]. To address this gap, mixture effect prediction approaches need to be integrated into insect pollinator risk assessment. Based on the mixture toxicity studies conducted here, it is evident that existing mixture hazard prediction models (CA and IA) can provide a first pass approach to assessment based on mode of action. Further, the ability to analyse mixture effects in pollinators using toxicokinetic and toxicodynamic DEBtox modelling approaches is demonstrated as a more comprehensive and mechanistically based approach that incorporates key physiological and resource allocation based traits into mixture effect assessment [40, 41].

For many chemical mixtures, with the possible exception of ergosterol biosynthesis inhibiting fungicides (see below), it is not fully established which combinations will operate in an additive manner according to the mode of action (i.e. similar, dissimilar) and which, if any show interactive joint effects (i.e. antagonistic, synergistic). This is the case for bees, as well as other species. While mixture testing to assess especially for synergism is not feasible for many mixtures, the testing of priority combinations is achievable. Among interactive mixtures, those that show synergism are the ones that may provide the greatest concern for regulators, as these have the possibility to result in joint effects in the field that would exceed those predicted based on information obtained from studies with the single chemical alone. Co-exposure involving sterol inhibiting fungicides have frequently shown synergism in a range of species [5, 24, 42, 43]. Already identified as synergistic in co-exposure with the pyrethroids tau-fluvalinate and lamba-cyhalothrin in honeybees [6, 35], the current study suggests such synergism albeit of small magnitude may be relevant to mixtures with neonicotinoids supporting previous findings by Iwasa et al [8]. Hence, such mixtures may be taken as a priority set of combinations for future mixtures studies to fully establish the extent of interaction across chemical combinations, different species and different exposure times. Further, by collating data to identify the range of joint mixture effects, probabilistic mixture assessment based on the frequency of deviation from mixture models can be derived. These would give the possibility of deriving robust protection criteria for pollinators, for exposure to chemical mixtures that still remain to be tested.
